# P-1671. Trends in Post-COVID Conditions Incidence Among Adults Aged ≥ 18 years: Temporal Changes in Affected Body Systems — United States, January 1, 2020 – June 30, 2024

**DOI:** 10.1093/ofid/ofaf695.1845

**Published:** 2026-01-11

**Authors:** Ndey Bassin Jobe, Caroline Pratt, Sharon Saydah

**Affiliations:** Centers for Disease Control and Prevention, Decatur, Georgia; Centers for Disease Control and Prevention, Decatur, Georgia; Centers for Disease Control and Prevention, Decatur, Georgia

## Abstract

**Background:**

Post-COVID Conditions (PCCs) are chronic conditions that occur following SARS-CoV-2 infection, are present for ≥ 3 months, and affect ≥ 1 body systems. Many patients have multiple PCCs. The risk of developing ≥ 1 PCC is higher in those who experienced severe COVID-19 illness or are unvaccinated. We describe the temporal changes in PCC incidence by most commonly affected body systems among adults from Tracking Post-COVID Conditions (Track PCC) network.

**Table 1:**
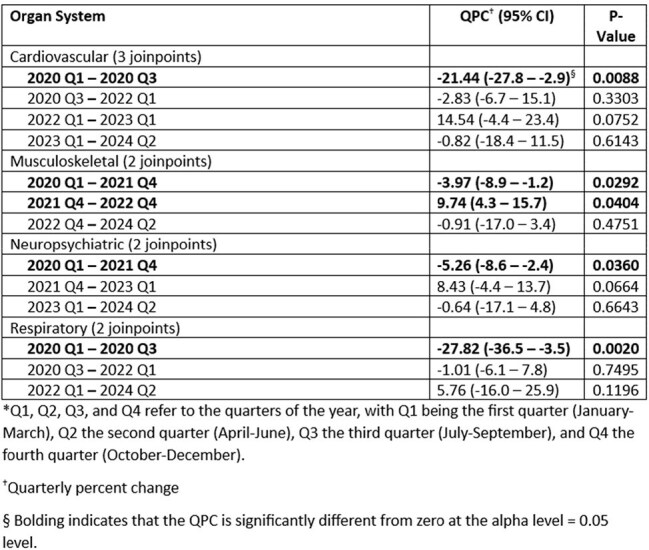
Quarterly* Trends in Crude Incidence Rates of Post-COVID Conditions (PCCs) — United States, January 1, 2020 – June 30, 2024

**Figure 1:**
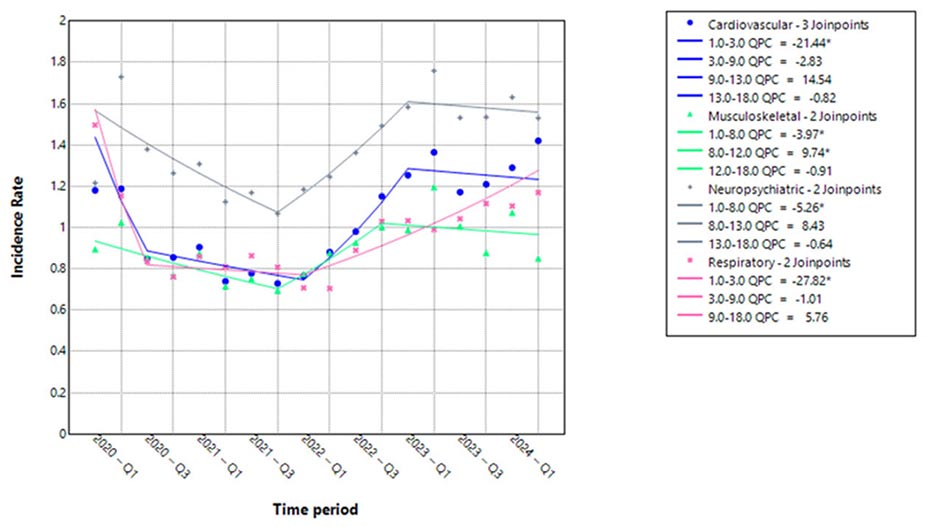
Crude Incidence Rates of Post-COVID Condition (PCC) and Joinpoint Models for Trends— United States, January 1, 2020 – June 30, 2024

**Methods:**

Electronic health data from Track PCC, a multi-state consortium with five surveillance sites across the U.S. Patients aged ≥ 18 years within each site’s catchment area January 2020–June 2024 were analyzed. Most commonly affected PCC body systems (cardiovascular, musculoskeletal, neuropsychiatric, respiratory) were assessed. Unadjusted incidence rates (IR) of PCC were calculated as the number of patients with ≥ 1 newly diagnosed PCC by body system within 31–90 days after confirmed acute COVID-19 illness among patients with ≥ 1 provider visits in the prior year and are reported per 1000 patient days. The standard errors (SE) of the IR were estimated using binomial variance approximation. To examine temporal trends, quarterly percent changes (QPCs) in IR were estimated, and points where trends changed significantly in direction or magnitude were identified using joinpoint regression.

**Results:**

Among the 190,465 adults who had ≥1 PCC, 59,315 (31%) adults had cardiovascular involvement, 56,142 (29%) had respiratory involvement, 85,309 (45%) had neuropsychiatric involvement, 54,893 had musculoskeletal involvement, and 51,783 (27%) had more than one of the four body systems involved.

PCC IR by body system decreased: in cardiovascular and respiratory (2020 Q1–Q3) by 21.44% and 27.82% (p = 0.0088 and 0.002, respectively); in musculoskeletal and neuropsychiatric (2020 Q1–2021 Q4) by 3.97% and 5.26% (p = 0.0292 and 0.036, respectively). PCC IR in musculoskeletal system (2021 Q4–2022 Q4) increased by 9.74% (p = 0.0404). From 2023–24, QPC did not change significantly (Table, Figure).

**Conclusion:**

PCCs continue to occur, and trends differ by body systems. Understanding the factors driving these trends may inform clinical and public health efforts to mitigate the impacts of PCC.

**Disclosures:**

All Authors: No reported disclosures

